# Simulated Gastrointestinal Digestion of Cocoa: Detection of Resistant Peptides and In Silico/In Vitro Prediction of Their Ace Inhibitory Activity

**DOI:** 10.3390/nu11050985

**Published:** 2019-04-30

**Authors:** Angela Marseglia, Luca Dellafiora, Barbara Prandi, Veronica Lolli, Stefano Sforza, Pietro Cozzini, Tullia Tedeschi, Gianni Galaverna, Augusta Caligiani

**Affiliations:** Department of Food and Drug, University of Parma, 43124 Parma PR, Italy; angela.marseglia@unipr.it (A.M.); luca.dellafiora@unipr.it (L.D.); barbara.prandi@unipr.it (B.P.); veronica.lolli@unipr.it (V.L.); stefano.sforza@unipr.it (S.S.); pietro.cozzini@unipr.it (P.C.); tullia.tedeschi@unipr.it (T.T.); gianni.galaverna@unipr.it (G.G.)

**Keywords:** cocoa, oligopeptides, simulated gastrointestinal digestion, angiotensin-converting enzyme (ACE) inhibitory activity

## Abstract

In this study we investigated the oligopeptide pattern in fermented cocoa beans and derived products after simulated gastrointestinal digestion. Peptides in digested cocoa samples were identified based on the mass fragmentation and on the software analysis of vicilin and 21 KDa cocoa seed protein sequences, the most abundant cocoa proteins. Quantification was carried out by liquid chromatography/electrospray ionisation mass spectrometry (LC/ESI-MS) using an internal standard. Sixty five peptides were identified in the digested samples, including three pyroglutamyl derivatives. The in vitro angiotensin-converting enzyme (ACE)-inhibitory activity of cocoa digests were tested, demonstrating a high inhibition activity, especially for digestates of cocoa beans. The peptides identified were screened for their potential ACE inhibitory activity through an in silico approach, and about 20 di-, three- and tetra-peptides actually present in our samples were predicted as active. Two of the potentially active peptides were chemically synthesized and then assessed for their inhibitory activity by using the ACE in vitro assay. These peptides demonstrated an ACE inhibitory activity, however, that was too weak to explain alone the high activity of cocoa digestates, suggesting a synergic effect of all cocoa peptides. As a whole, results showed that an average chocolate portion (30 g) ensures an amount of peptides after digestion that, assuming complete absorption, could reach almost a complete inhibition of ACE.

## 1. Introduction

Cocoa beans, from the fruit of the cocoa tree (*Theobroma cacao L*.), are transformed into chocolate and other cocoa products by a complex process involving fermentation, drying and roasting. By the 1600s and 1700s, chocolate and cocoa were viewed not just as a beverage with a pleasurable taste, but also as a food to treat a number of disorders [1]. Possible health benefits of chocolate have been reported for many years but it is only recently that some of these claims are being more clearly identified and studied. For example, the antioxidant and health-promoting properties of cocoa and cocoa-related products have been thoroughly investigated and various health claims for cocoa polyphenolics have been proposed [2]. Recently, the European Food Safety Authority (EFSA) issued a positive opinion on cocoa flavanols and maintenance of endothelium-dependent vasodilation, which contributes to normal blood flow [3]. While the polyphenols and antioxidant activity of cocoa have been extensively studied, little is known about the potential health effects of other cocoa components such as peptides/proteins [4]. Peptides are currently considered important bioactive constituents of food, however the potential biological activities of oligopeptides found in cocoa are under-investigated in cocoa literature. Biologically active or functional peptides are food-derived peptides that exert, beyond their nutritional value, a physiological effect in the body [5]. Dietary proteins provide a rich source of bioactive peptides, which are hidden in a latent state within the native protein, requiring enzymatic proteolysis for their release. Bioactive peptides can be produced during in vivo gastrointestinal digestion and/or food processing and they have been reported in a wide range of animal and vegetable proteins, such as bovine and human milk, fish, meat, soybean and cereals [6]. In vitro and in vivo studies demonstrated several biological functions attributed to bioactive peptides, such as antimicrobial, immunomodulatory, enhancement of mineral absorption, antithrombotic, antihypertensive, opioid and antioxidant activities [7].

In cocoa, peptides are naturally formed during cocoa beans’ fermentation, as previously reported [8,9,10,11], and together with amino acids they are considered important flavour precursors [12,13,14]. Peptides in cocoa derive from the two major protein fractions, globulins, consisting of a 66 kDa vicilin-like storage protein [15], and albumin consisting of a 21 kDa protein with trypsin inhibitory properties [16]. Cocoa proteins during natural cocoa fermentation are cleaved to hydrophilic and hydrophobic peptides as well as amino acids through autolysis by two endogenous enzymes, aspartic endoprotease and carboxypeptidase activated by microbial metabolites as acetic acid [17]. Besides generating the characteristic chocolate aroma, the potential biological activities of oligopeptides found in cocoa are of interest. Two papers report the physiological effects (antioxidant, angiotensin-converting enzyme inhibitors and hypoglycaemic activities) of cocoa autolysates containing peptides and amino acids [18,19] and, more recently, the antioxidant properties of cocoa protein enzymatic hydrolysates [20]. A patent for the production of angiotensin-converting enzyme (ACE) inhibitory peptides from cocoa was also developed [21], demonstrating the growing interest in the topic. However, the identification of peptides responsible for the activities is largely unknown, together with the resistance of peptides through the gastrointestinal tract, a pre-requisite for their bioavailability. In fact, before testing any systemic biological activity of food peptides mixtures, it is of utmost importance to assess their bioavailability, and as a first step their resistance to gastrointestinal digestion. Moreover, gastrointestinal digestion might even form new peptides, with more biological activity. However, the information about the peptide composition of cocoa after ingestion is, at the moment, lacking in the literature. Therefore, the aim of this study was the identification of the peptides released after in vitro simulated gastrointestinal digestion of cocoa beans and derived products, using a physiological digestion model. Moreover, the potential ACE inhibitory activity of cocoa peptides found after digestion was evaluated by an in silico/in vitro combined approach.

## 2. Materials and Methods

### 2.1. Cocoa Samples

The release of oligopeptides by in vitro gastrointestinal digestion model was investigated for different typologies of cocoa samples. Two samples of well fermented cocoa beans of Forastero varieties of different geographical origins (Congo and Dominican Republic) were kindly provided by Barry Callebaut, Belgium. To highlight the eventual different release of peptides due to the cocoa process, one intermediate product of the cocoa processing chain (cocoa paste) and one end product (dark chocolate bar, 40% of cocoa mass) were also included in the experimental plan.

### 2.2. Extraction of Peptides from Non-Digested Samples

Peptides were extracted according to the method previously described [8]. A total of 10 g of finely grinded cocoa sample was suspended in 45 mL of 0.1 N HCl. (L,L)-phenylalanylphenylalanine (Phe-Phe) was added as an internal standard (2.25 mL of a 1 mM solution). The suspension was homogeneized for 1.5 min by Ultra Turrax T50 at 4000 rpm (Janke and Hunkel Labortechnik, Germany) and then centrifuged at 4000 rpm for 30 min at 4 °C by an ALC 4237R centrifuge. The solution was filtered through paper filters (pore dimensions 15–20 µm) and then extracted four times with 50 mL of ethyl ether. The solution was filtered again with a Millipore 47 mm Steril Aseptic system through 0.45 µm HVLP millipore filters. A total of 1.5 mL of the resulting solution were mixed with 0.5 mL of a formic acid solution (0.1%). The solution was diafiltered through Sartorius Vivaspin 2 filters (nominal molecular cut-off 10 KDa) by using an Amicon Micropartition system MPS-1. The filtrate was dried under nitrogen, redissolved in water with formic acid (0.1% *v*/*v*), and analyzed by ultra-high performance liquid chromatography/electrospray ionisation mass spectrometry (UPLC/ESI-MS).

### 2.3. Simulated In Vitro Gastro-Intestinal Digestion

Following the procedure described by Minekus et al. [22], 2.5 g of finely ground cocoa were digested. Briefly, three main steps were carried out: salivary phase, gastric phase and intestinal phase. 3.5 mL of simulated salivary fluid (15.1 mM KCl, 3.7 mM KH_2_PO_4_, 13.6 mM NaHCO_3_, 0.15 mM MgCl_2_ and 0.06 mM (NH_4_)_2_CO_3_) were added to the sample, together with 0.5 mL of amylase solution (1500 U/mL), 25 µl of calcium chloride (300 mM) and 0.975 mL of distilled water. Samples were briefly stirred using a vortex and incubated for 2 min at 37 °C on a reciprocating shaker (Stuart Scientific, Staffordshire, UK). Then, 7.5 mL of simulated gastric fluid (6.9 mM KCl, 0.9 mM KH_2_PO_4_, 25 mM NaHCO_3_, 47.2 mM NaCl, 0.1 mM MgCl_2_ and 0.5 mM (NH_4_)_2_CO_3_) were added to the samples, together with 1.6 mL of pepsin solution (25,000 U/mL), 5 µL of calcium chloride (300 mM), 0.2 mL of 1 M hydrochloric acid and 0.695 mL of distilled water. The pH was adjusted to 3 with 1 M HCl and the samples briefly stirred using a vortex and incubated for 2 h at 37 °C on a reciprocating shaker (Stuart Scientific, Staffordshire, UK). Finally, 11 mL of simulated intestinal fluid (6.8 mM KCl, 0.8 mM KH_2_PO_4_, 85 mM NaHCO_3_, 38.4 mM NaCl and 0.33 mM MgCl_2_) were added, together with 5 mL of pancreatin solution (800 U/mL), 2.5 mL of bile solution (75 mg/mL), 400 µL of calcium chloride (300 mM), 150 µL of 1 M sodium hydroxide and 1.31 mL of distilled water. The pH was adjusted to 7 using NaOH 1 M and the samples briefly stirred using a vortex and incubated for 2 h at 37 °C on a reciprocating shaker (Stuart Scientific, Staffordshire, UK). To stop the digestion and inactivate enzymes, samples were heated at 100 °C for 10 min.

After cooling, 1 mL of the internal standard Phe-Phe (1 mM) is added and the solution acidified by HCl (pH 1–2). Samples are centrifuged for 30 min at 4000 rpm at 4 °C to precipitate insoluble proteins and undigested compounds, then the aqueous phase is separated and subjected to two extractions with diethyl ether to remove lipids and then treated as previously reported for undigested samples.

### 2.4. Ultra-High Performance Liquid Chromatography/Electrospray Ionisation Mass Spectrometry (UHPLC/ESI-MS) Analysis Conditions

Peptides were analyzed by a UHPLC/ESI-MS system (ACQUITY Ultra Performance LC, WATERS, Milford, MA, USA) in the following conditions. Eluent A: water with 0.1% (*v*/*v*) formic acid and 0.2% (*v*/*v*) acetonitrile; eluent B acetonitrile with 0.1% (*v*/*v*) formic acid; gradient elution was performed according to the following steps: 0–7 min isocratic 100% A, 7–50 min linear gradient from 100% A to 50% A, 50–52 min isocratic 50% A, 53–58 min from 50% A to 0% A and reconditioning. Column: AQUITY UPLC BEH C18 (1.7 µm, 2.1 mm × 150 mm). Column temperature was 35 °C. Injection volume was 2 μL; flow rate: was 0.2 mL/min. MS conditions: ESI, positive ions, single quadrupole analyzer. Capillary voltage: 3.2 kV; cone voltage: 30 V; source temperature: 150 °C; desolvation temperature: 300 °C; cone gas flow (N2): 100 L/h; desolvation gas (N2): 650 L/h; acquisition: 100:2000 *m*/*z*. All data were acquired and processed by the software MassLynx 4.0 (Waters, Milford, MA, USA).

### 2.5. Peptides Identification by High-Performance Liquid Chromatography/Tandem Mass Spectrometry (HPLC/MS-MS)

Low-resolution mass spectrometry (LRMS) analysis was performed on digested cocoa samples, in order to identify the amino acid sequences of the peptides. LRMS analysis was performed in positive mode accordingly to Prandi et al. [23]. Prior to LC-MS analysis, samples were centrifuged at 6708× *g*, 4 °C for 10 min to precipitate insoluble compounds. Chromatographic separation was achieved using a reverse phase column (Aeris Peptide 1.7 µm XB-C18, 150 mm × 2.10 mm, Phenomenex, Torrance, CA, USA) in an UHPLC system (Dionex Ultimate 3000, Thermo Scientific, Waltham, MA, USA). Eluent A was water with 0.1% (*v*/*v*) formic acid and 0.2% (*v*/*v*) acetonitrile, eluent B was acetonitrile with 0.1% (*v*/*v*) formic acid and 0.2% water. Flow was maintained at 0.2 mL/min and the gradient applied was: 0–7 min, 100% A; 7–50 min, from 100% A to 50% A; 50–52.6 min, 50% A; 52.6–53 min, from 50% A to 0% A; 53–58.2 min, 0% A; 58.2–59 min, from 0% A to 100% A; 59–72 min, 100% A. Total run time: 72 min; column temperature: 35 °C; sample temperature: 18 °C; injection volume: 2 µL. Detection was achieved using a triple quadrupole TSQ Vantage (Thermo Scientific, Waltham, MA, USA) using the following parameters: positive ion mode, acquisition time: 7–58.2 min (7 min of solvent delay were applied at the beginning of the chromatographic run), acquisition range: 100–1500 *m*/*z*; micro scans: 1; scan time: 0.50; Q1 PW: 0.70; spray voltage: 3200 V; capillary temperature: 250 °C; vaporizer temperature: 250 °C; sheath gas flow: 22 units. Different collision energies (CE) were applied depending on the mass and charge of the ion to be fragmented. Peptides fragments were also compared with cocoa peptide sequences reported by D’Souza et al. [11].

### 2.6. Peptides Quantification

Peptides from digested and undigested samples were quantified by comparison to the internal standard (Phe-Phe), assuming a response factor equal to 1. For the correct integration of peaks, the extract ion chromatogram (XIC) technique was applied. The oligopeptides were semi-quantified by measuring the ratio between the XIC peptide area and the relative XIC area of Phe-Phe, as previously described [24]. Data obtained were expressed as mg of peptide respect to cocoa sample assuming that the specific response factor for each peptides is equal to 1, which is certainly not the case. With this limitation in mind, the quantitative data were used mostly for comparative purposes and to highlight trends and differences among samples.

### 2.7. Determination of In Vitro Angiotensin-Converting Enzyme (ACE) Inhibitory Activity of Cocoa Digestates

The percentage of ACE inhibitory activity for digested cocoa samples was determined by using the methods of Cushman et al. [25] with some modifications as reported by Dellafiora et al. [26] and according to the following equation:

I% = [(ACEmax − Bmax) − (ACEmin − Bmin)]/(ACEmax − Bmax) × 100(1)

where ACEmax is the maximum activity of ACE (in the absence of the cocoa peptides), ACEmin is the minimal activity of ACE (in the presence of the peptides), Bmax is the control blank of ACE and Bmin is the control blank of sample/pure peptide.

The following solutions were prepared: sodium borate buffer (0.1 M, NaBB) with NaCl (300 mM), pH 8.3; potassium phosphate buffer (0.01 M, KPB) with NaCl (500 mM), pH 7; 5 mM hippurylhistidyl-leucine (HHL) in NaBB buffer; and ACE 0.1 U/mL in KPB + 5% glycerol (g/mL). The experiment was carried out at 37 °C in a thermostatic bath with the following parameters: maximum activity of ACE (ACEmax) = 200 μL of HHL + 80 μL of digestion blank + 20 μL of ACE; control blank of ACE (Bmax) = 200 μL of HHL + 80 μL of NaBB + 20 μL of KPB; minimal activity of ACE (ACEmin) = 200 μL of HHL + 80 μL of sample (i.e., fraction or pure peptide) + 20 μL of ACE; control blank of sample (i.e., fraction or pure peptide) (Bmin) = 200 μL of HHL + 80 μL of sample (i.e., fraction or pure peptide) + 20 μL of KPB. After 60 min of incubation, the reaction was quenched with 250 μL of HCl (1 N).

Because the digested cocoa extract is a complex mixture containing several compounds, each of which could possess a potential ACE inhibitory activity, solutions containing compounds representative of the main cocoa molecular classes present in the extracts (theobromine for methylxanthines, epicatechin for polyphenols and free amino acids) and simulating the real concentrations contained in the cocoa extract were also prepared. The analysis of HHL and hyppuric acid (HA) was performed by UPLC-ESI-MS in the following conditions: eluent A: H_2_O (0.2% CH_3_CN and 0.1% HCOOH); eluent B CH_3_CN (0.1% HCOOH); gradient elution was performed according to the following steps: 2 min isocratic 100% A, 2–6 min linear gradient from 100% A to 0% A and reconditioning. Column: AQUITY UPLC BEH C18 (1.7 µm, 2.1 mm × 150 mm). Flow rate: 0.25 mL/min. MS conditions: ESI, negative ions, single quadrupole analyzer. Capillary voltage: 2 kV; cone voltage: 30 V; source temperature: 150 °C; desolvation temperature: 300 °C; cone gas flow (N2): 100 L/h; desolvation gas (N2): 650 L/h; acquisition: 80:1000 *m*/*z*. ACE activity was determined by calculating the ratio of HA and HHL, utilizing as quantification ions *m*/*z* 178 and 428 respectively.

Antihypertensive activity was determined on cocoa digestate NaBB solutions, containing 50 mg cocoa mass/mL, corresponding to an approximate range of peptides of 10–30 μg/mL (quantitative amount are reported in Table 1).

### 2.8. Prediction of ACE Inhibitory Activity of Cocoa Digestate Peptides by Computational Procedures

#### 2.8.1. Pharmachopore Models

The anatomy of the open and closed ACE binding sites was investigated by using the Flapsite tool of FLAP software (Fingerprint for Ligand And Protein; http://www.moldiscovery.com, Hertfordshire, UK) [27], and the GRID molecular interaction fields (MIFs) was used to investigate the corresponding pharmacophoric space. The DRY probe was used to describe the potential hydrophobic interactions, while the sp2 carbonyl oxygen (O) and the neutral flat amino (N1) probes were used to describe the hydrogen bond donor and acceptor capacity of the target, respectively. All images were obtained using the software PyMol version 1.7 (http://www.pymol.org, Schrodinger, LLC, New York, NY, USA).

#### 2.8.2. Molecular Modelling

The models for both C- and N-domains of ACE were derived from the Protein Data Bank (http://www.rcsb.org) structures having PDB codes 4APH and 4BZS, respectively. Protein structures and ligands were processed by using the software Sybyl, version 8.1 (www.tripos.com, Certara USA, Inc., Princeton, NJ, USA). All atoms were checked for atom- and bond-type assignments. Amino- and carboxyl-terminal groups were set as protonated and deprotonated, respectively. Hydrogen atoms were computationally added to the protein and energy-minimized using the Powell algorithm whit a coverage gradient of ≤0.5 kcal (mol Å)–1 and a maximum of 1500 cycles.

Anatomy of the pocket. The two catalytic domains of ACE originated from tandem gene duplication (ref) and maintain the same 3D organization with 51% of sequence identity (according to global alignment by using the Needleman–Wunsch algorithm; http://www.ebi.ac.uk/Tools/psa/emboss_needle). Both domains hold a huge pocket with similar shape which crosses the entire protein body. However, analyses were focused on catalytic sites retracing the mode of action of inhibitory drugs (Yates et al., 2014). The regions lining the catalytic site maintain the same organization in both domains and both pockets share a prevalently hydrophobic environment, albeit they differ for 7 amino acid substitutions.

#### 2.8.3. Docking Simulations and Rescoring Procedure

The coupling of GOLD, to perform docking simulations, and HINT (Hydrophatic INTeraction) software, as re-scoring function, has been already proved to be effectively able to evaluate the bioactivity of small molecules [28,29,30,31] including peptides [32]. The docking simulations of compounds were performed with the GOLD version 5.1 (CCDC, Cambridge, UK; http://www.ccd.cam.ac.uk). All crystallographic waters and ligands were removed and 25 poses for each compound were generated. No constraints were set up, and the explorable space was defined in a radius of 10 Å from the centroid of the catalitic site. For each GOLD docking search, a maximum number of 100,000 operations were performed on a population of 100 individuals with a selection pressure of 1.1. Operator weights for crossover, mutation and migration were set to 95, 95, and 10, respectively. The number of islands was set to 5 and the niche to 2. The hydrogen bond distance was set to 2.5 Å and the van der Waals linear cut-off to 4.0. Ligand flexibility options “flip pyramidal N”, “flip amide bonds”, and “flip ring corners” were allowed. Each best scored pose according to GOLD scoring function was re-scored by HINT. Owing to the huge dimension of the pocket, the molecules’ positioning has been spatially restrained according to crystallographic pose of the inhibitory drug captopril.

The software HINT [33] was used as the re-scoring function on the basis of previous studies attesting the higher reliability of HINT scoring with respect to other scoring functions, as well as of its successful use in the search for ligands for other targets and in the estimation of ligand binding free energies. In more detail, the score provides the evaluation of thermodynamic benefits of protein-ligand interaction, and therefore low/negative scores indicate not appreciable protein-ligand interactions ([29,30,31,32,34]). GOLD uses a Lamarckian genetic algorithm and scores may slightly change from run to run. Therefore, in order to exclude a non-causative score assignment, we conducted simulations in quintuplicate and the mean values are reported.

#### 2.8.4. Chemical Synthesis of Specific Peptides Predicted as Active

Peptides VPI and SPV were synthesized on solid phase according Fmoc/t-butyl strategy using a Syro I Fully Automated Peptide Synthesizer (Biotage, Uppsala, Sweden). The peptides were cleaved from the Wang resin using a TFA:TIS:H_2_O (95:2.5:2.5) solution, precipitated with diethyl ether and desalted on Sep-Pack C18 cartridges (Waters Corporation, Milford, MA, USA). Characterization MH^+^ (ESI–MS): 328.29 VPI, 302.15 SPV. Each peptide was tested as reported above for its ACE inhibitory activity, using 80 μL of NaBB solution as blank sample for ACEmax. In this case, the specific IC50 was also calculated. The IC50 value is defined as the inhibitor concentration that is able to decrease ACE activity by 50%. To determine IC50, different concentrations of peptides were prepared and their relative ACE inhibitory activity was evaluated. IC50 values were determined by plotting the percentage relative inhibition as a function of concentration of test compound.

## 3. Results and Discussion

The gastrointestinal digestion process has an influence not only on the hydrolysis of peptides still present in food, but also on the release of peptide sequences encrypted in food proteins. In order to have a complete picture of the fate of cocoa peptides during the digestion process, both those already present and those released by the proteins, different cocoa samples were analysed before and after simulated gastrointestinal digestion. In particular, fermented cocoa beans (currently used as healthy food or as ingredients in cocoa and bakery products), cocoa paste and dark chocolate were considered. The digestion procedure was adapted from Minekus et al. [22]: the method mimics the subsequent steps of the digestion process in terms of composition of the juices in the different compartments (simulation of salivary juice, gastric juices, duodenal juice and bile) as well as the relative residence times.

**Figure 1:**
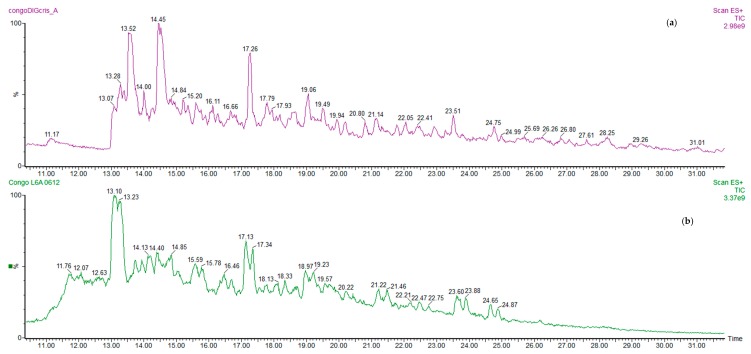
Ultra-high performance liquid chromatography/electrospray ionisation mass spectrometry (UPLC/ESI-MS) chromatogram of a digested sample of Congo fermented cocoa bean (**a**) compared to the peptide profile of the corresponding not digested, extracted sample (**b**).

Typical MS chromatograms (full scan acquisition) of a cocoa bean sample of Congo origin are shown in Figure 1, showing the aqueous acidic extract containing peptides, obtained before digestion, compared with that of the digesta.

**Figure 2:**
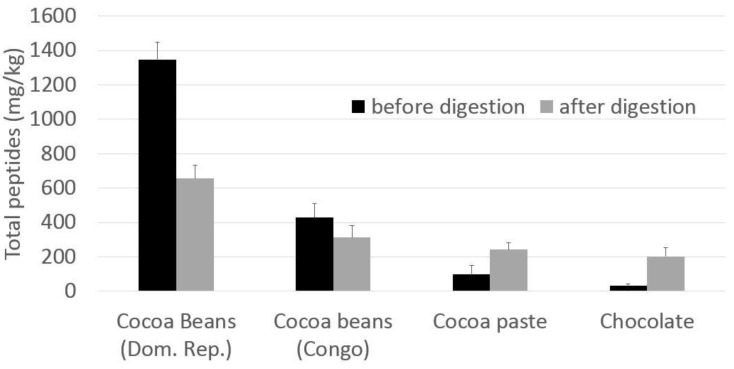
Peptides amount (mg/kg) in digested sample compared to the amount in the respective non-digested samples. Peptides were quantified as ratio of peptides area vs. internal standard area (phe-phe) and referred to the initial cocoa amount, making the raw assumption that the specific response factor for each peptides is equal to 1. The total peptides amount is referred to the sum of peptides listed in Table 1.

The quali-quantitative peptide profile of digesta from fermented cocoa beans is quite different with respect to that of the extract before digestion. The main peptides in the chromatograms were identified on the basis of data previously reported [8,9] on undigested cocoa beans, and some new peptides were further identified by HPLC/ESI-MS-MS. The complete list of peptides identified is reported in Table 1. In Figure 2 the amounts of total peptides in samples before and after digestion were compared, in order to better understand the formation/degradation of peptides in simulated digestion. Results showed that gastrointestinal digestion has different effects depending on the cocoa products: total peptide amount in fermented cocoa bean digested samples is generally reduced with respect to the corresponding non-digested samples (Figure 2), while in cocoa products (cocoa paste and chocolate) the amount of peptides released during digestion and resistant to the protease activities is higher respect the amount before digestion. This behaviour could be to the effect of thermal treatment occurring in cocoa beans to produce cocoa paste and chocolate. In the case of chocolate, it has to be taken into account also that other ingredients are added, such as sucrose, cocoa butter, lecithin etc. which probably have an effect on peptide bioaccessibility from proteases.

As far as the original peptides present in cocoa, many peptides seem to resist to simulated gastrointestinal digestion, even if to different extent. Peptides showing the higher decrease upon digestion are DVF, GDVF, IEF, PGDVF, SPGDVF, KDQPL, DEEGNFKIL, all having Phe or Leu as C-terminal. Some peptides are most abundant in digested samples respect to undigested, especially in cocoa-derived products (cocoa paste and chocolate), indicating that they are formed during gastrointestinal digestion. The peptides released are mainly dipeptides, e.g., II, LL, LI, IL, PI, PL, PI, VI, VL, AI. This behavior is in line with those of other food matrices, both animal and vegetal, as for example raw ham, cheese, soybean, which are recognized as a source of bioactive peptides mainly formed during digestion [35,36,37,38].

The pattern of resistant peptides after in vitro gastrointestinal digestion is of particular significance for the eventual bioactivity of a cocoa nitrogen fraction.

### 3.1. Potentially Bioactive Peptides

We identified for the first time the sequences of peptides derived from cocoa protein resistant to (or formed during) in vitro gastrointestinal digestion and, as a consequence, potentially bioaccessible, so it could be of interest to evaluate their potential bioactivity. It is known that in order to stimulate a biological response, the peptides must be bioavailable, i.e., following digestion, they must be able to cross the intestinal epithelial cells and enter the blood circulatory system, or produce local effects in the gastrointestinal tract [39]. The question of the absorption of oligopeptides is paramount to the science of food-derived bioactive peptides and it has been recently reviewed [40]. Although the complete mechanism of absorption and the bioavailability of the specific peptides were not investigated, there is some evidence that small food bioactive peptides are bioavailable and can be absorbed into the body [41]: whereas there is evidence for some uptake of intact di- and tripeptides from the human gastrointestinal tract, such uptake is not ubiquitous and there is little support for the uptake of tetrapeptides and larger peptides. Moreover, it is not completely clear what are the effects of cytosolic, vascular endothelial tissue peptidases and soluble plasma peptidases, and the half-life of many peptides in the plasma seems to be very short. Despite all these considerations on the limited evidence for the effective health effects of peptides in humans, the constant growthe of literature concerning bioactive peptides in food is a testament to the large interest in this field from the scientific community. Therefore, it has resulted in interest in predicting the potential bioactivity of new peptide sequences from new food sources, as in the case of cocoa.

As a first approach, we evaluated the bioactive potential of cocoa peptide formed during digestion using the BIOPEP database (http://www.uwm.edu.pl/biochemia/index.php/pl/biopep). Because no studies are present in literature reporting the sequences of bioactive peptides in cocoa, many of the peptides found in this work are not listed in the BIOPEP database. However, some cocoa peptide sequences, mainly dipeptides, found in digested samples, match sequences reported in the BIOPEP database, and their predicted activities are reported in Table 1. Cocoa samples showed high occurrence of sequences of angiotensin-converting enzyme-inhibitor peptides (ACE) as well as of peptides having glucose uptake stimulating activity and dipeptidyl peptidase IV inhibitor, suggesting some possible activities on blood pressure and diabetes, in agreement with the results of the previous works reporting the antioxidant, ACE inhibitory and hypoglycaemic activities of cocoa autolysates [18,19].

### 3.2. ACE Tests on Cocoa Digested Samples

Among the possible bioactivity of cocoa peptides, we focused on the angiotensin I converting enzyme (ACE) inhibitory activity, due to the beneficial effects of ACE inhibitory peptides on hypertension, representing one of the major risk in cardiovascular diseases [42]. ACE is an extracellular enzyme expressed in many types of endothelial cells, especially in the capillaries of the lung, as well as in epithelial cells in the kidney, small intestine and epididymis [43]. Bioactive peptides are currently viewed as a nutraceutical approach to moderately control the blood pressure level, even as prevention in healthy subjects, without bringing a sharp decrease of the pressure or other side effects.

The aqueous peptide fractions of digestate of cocoa bean samples and chocolate were tested for their ACE inhibitory activities. The digestates tested contained 50 mg/mL of cocoa sample, corresponding to an approximate range of peptide amount of 10–30 μg/mL, as resulted from the quantitative analysis of cocoa peptides in digestates reported in Table 1.

Because the aqueous fraction of cocoa digestate contained also significant amounts of other cocoa hydrosoluble compunds (mainly free amino acids, epicatechin and theobromine), separate solutions (model systems) of theobromine, epicatechin and a mixture of amino acids in the proportion found in cocoa proteins were prepared, with concentrations simulating those actually present in the digested fractions of cocoa products. These solutions were tested for their specific ACE inhibitory activity.

From the results of ACE inhibition (I%) reported in Table 2, it is possible to highlight that the activity of the ACE remains very high in the presence of the mixture of AA, theobromine and epicatechin, indicating that these components have a weak ACE inhibitory activity and do not significantly contribute to the activity that follows registered for cocoa samples.

Instead, the activity of ACE was strongly inhibited when cocoa beans, cocoa paste and chocolate were tested, indicating a strong ACE inhibitory activity especially for cocoa beans. This is coherent with the higher amount of peptides present in cocoa bean solution with respect to cocoa paste and chocolate, confirming that the peptide fraction is the most active against ACE. Consuming fermented beans is not so common, even if some products start to appear on the market (for example some cookies containing a part of cocoa beans), due to the higher polyphenol amount and, therefore, the better health properties. Anyway, the percentage of inhibition reached is higher than 50% (IC_50_) for all the cocoa samples tested, comprising chocolate, representing a common cocoa product consumed all over the world. Considering that the 50% inhibition is obtained in this work digesting 2.5 g of cocoa beans/cocoa products, it is evident that it is possible to obtain a significant concentration of cocoa peptide by consuming a normal cocoa portions (30 g, as reported by LARN (Livelli di Assunzione Raccomandata di Nutrienti per la popolazione italiana, 2014 [44]). Therefore, one can hypothesize that physiologically relevant concentrations might be reached after dietary consumption of cocoa products, at least in the intestinal lumen.

### 3.3. In Silico Screening of Potential ACE Inhibitory Peptides

To go deeper inside into the molecular mechanisms of cocoa ACE inhibition, we proceeded to test the specific bioactivity of each resistant cocoa peptide identified in the digestates using a computational in silico approach. The principal advantage of using this approach is the reduction of the experimental efforts in the early stage of bioactive peptides identification, avoiding matrix isolation, chemical synthesis and an in vitro or in vivo bioactivity test of non-active peptides. Utilizing as a first screening the in silico approach used in this research, it is possible to assign a priority ranking of peptides in terms of ACE-inhibitory activity, with the final aim to guide the choice of peptides to be synthesized and then assessed for their inhibitory activity by using ACE in vitro assay.

ACE is a zinc-dependent carboxypeptidase organized in two distinct catalytic domains. Both hydrolyze substrates by removing one or more dipeptides from the C-terminal end. In particular, antihypertensive activity is based on the ability of several peptides to inhibit ACE, which in vivo converts the decapeptide angiotensin I into the octapeptide angiotensin II, the latter being able to induce vasoconstriction and to increase blood pressure Computational prediction of peptides ACE inhibitory activity is based on the assumption that the protein–ligand interaction is the condition for inhibitory activity. Consequently, in the case of ACE, a peptide is considered active if it is able to interact with at least one of the two domains. Therefore, the coupling of docking simulations and rescoring procedures by using the HINT scoring function—whose correlation with the free energy of binding was previously reported [34]—can be used effectively to predict the inhibitory activity.

Among the entire peptide profile identified after the in vitro physiological digestion of cocoa beans and derived products (Table 1), we focused only on low Mw peptides (di-, tri- and tetrapeptides) for the computational analysis. This inclusion criterion was based on the finding that short peptides may be easily adsorbed trough the intestinal epithelium [41], thus giving a greater physiological significance. The computational results obtained are reported in Table 3, where 19 peptides were predicted as active, 17 in both C-domain and N-domain of ACE, and 2 peptides that were active in only one domain (LLDR in N-domain and QLGN in C-domain).

### 3.4. ACE in Vitro Test on Pure (Synthetized) Peptides

**Figure 3:**
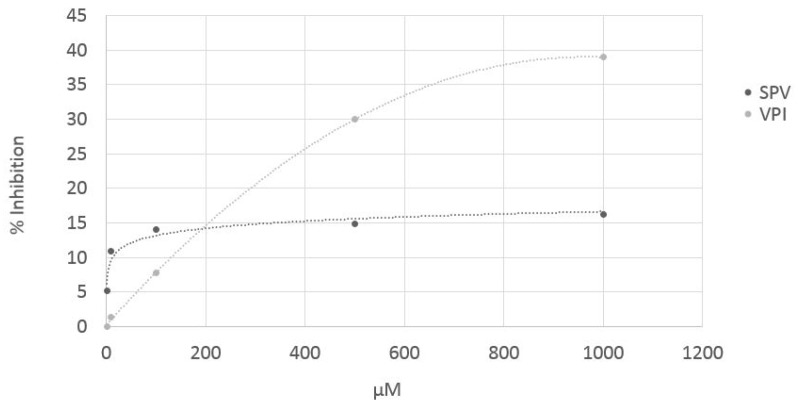
Sigmoid curves obtained by plotting the inhibitory activity of pure peptides SPV and VPI (synthetized by Fmoc protocol) against peptide concentration.

Two cocoa tripeptides predicted as active by computational procedure (VPI and SPV) were chemically synthetized by the Fmoc protocol on an automatic solid-phase peptide synthesizer (Syro I, Biotage) and tested for their effective in vitro ACE inhibitory activity. Cocoa dipeptides predicted as active were not synthetized and tested because their activity was in most cases still known in literature (BIOPEP database). ACE inhibitory activity of pure peptides VPI and SPV was determined by a UPLC/ESI-MS method, injecting the reaction products of ACE and hyppuryl-hystidyl-leucine as substrate, in presence and in absence of peptides. Both peptides predicted as ACE inhibitors by in silico approach, resulted inhibitors also in vitro. However, they are very weak inhibitors, because the IC50 is still not reached at concentrations higher than 1000 µM (Figure 3). These data suggest that the activity registered for cocoa samples is probably the effect of the synergic action of the whole peptide profile and, in particular, also the di-peptide pattern, could contribute significantly to the effect.

## 4. Conclusions

The research methodology utilized in this paper poses the bases for the determination of cocoa peptides’ bioactivity, a topic largely under-investigated with respect to other food peptides and also with respect to other cocoa components (e.g., polyphenols). Cocoa peptide mixtures obtained after simulated gastrointestinal digestion are able to strongly reduce in vitro ACE activity. Moreover, the in silico approach resulted in an effective tool in prediction of cocoa peptides’ bioactivity, giving the opportunity to reduce the experimental work aimed at the synthesis of peptides and bioactivity tests. However, the cocoa tri-peptides predicted as more active, synthetized and effectively tested for their in vitro bioactivity, resulted in being weak inhibitors, suggesting that probably the dipeptide pattern is the main one responsible for the activity registered for cocoa samples. A reasonable amount of the average chocolate portion (30 g) ensures a quantity of peptides after digestion that, assuming complete absorption, could reach almost a complete inhibition of ACE.

This study further supports the notion that cocoa could be an important component of a healthy diet by preventing chronic diseases. Taken as a whole, our findings are an important step forward to the in depth molecular characterization of such a relevant food product beyond its well-known nutritional properties.

## Figures and Tables

**Table 1:** Peptides identified in cocoa and derived products before and after digestion, semi-quantitative amounts (mg/kg cocoa) and reported bioactivity from BIOPEP database. Quantitative amounts are calculated utilizing the ratio of peptide area vs. internal standard area (phe-phe), assuming that all response factors are equal to 1.

Retention Time (min)	MH^+^	Sequence	Cocoa Protein (Vicilin, V; Albumin, 21k)	Bioactivity	Cocoa Bean (Congo) before Digestion	Cocoa Bean (Congo) after Digestion	Cocoa Bean (Dominican Rep.) before Digestion	Cocoa Bean (Dominican Rep.) after Digestion	Cocoa Paste before Digestion	Cocoa Paste after Digestio	Chocolate before Digestion	Chocolate after Digestion
10.62	203.2	AI	v	Angiotensin-converting enzyme (ACE) inhibitor	35.8	6.0	6.8	2.2	5.3	5.9	1.7	4.1
12.99	403.4	RLD	21k		2.2	0.0	11.7	1.4	0.5	0.3	0.1	0.3
13.02	295	FE			7.8	5.0	41.4	21.6	2.2	4.0	0.8	3.1
13.13	485.3				4.0	0.2	43.7	0.3	2.8	0.2	0.6	0.0
13.23	302	SPV			4.9	3.8	29.6	21.1	0.4	2.6	0.2	1.5
13.26	290.2	GTI	v		0.2	1.6	1.5	0.2	0.2	1.9	0.0	2.1
13.35	237.2	MS			1.4	1.3	19.4	8.8	1.0	1.6	0.4	1.0
13.42	281.2	DF		ACE inhibitor	3.1	4.4	28.4	14.1	1.6	4.2	0.6	3.4
13.5	223.2	FG	V	ACE inhibitor (FG)	14.2	5.6	51.4	27.3	3.5	4.2	1.2	2.8
13.74	229.3	PI (L)	21k/v	dipeptidyl peptidase IV inhibitor (PI, PL); ACE inhibitor (PL)	4.0	7.1	20.3	12.7	2.3	12.0	0.8	13.3
13.81	231.2	L (I) V	v/21k	glucose uptake stimulating peptide (IV, LV); dipeptidyl peptidase IV inhibitor (LV)	13.7	17.4	2.0	0.0	2.0	27.0	0.7	30.2
14	276.2	QE		dipeptidyl peptidase IV inhibitor	9.1	6.1	28.2	8.9	2.6	1.7	1.1	1.3
14.35	761.4	RRSDLD	21k		0.0	0.3	0.3	0.4	0.0	0.3	0.0	0.3
14.35	231.2	VI (L)	v/21k	dipeptidyl peptidase IV inhibitor (VI, VL), glucose uptake stimulating peptide (VL)	8.2	7.1	32.7	26.1	1.9	6.1	0.7	5.3
14.45	223.2	GF		ACE inhibitor, dipeptidyl peptidase IV inhibitor	3.5	4.0	0.0	4.2	0.3	5.8	0.1	6.7
14.64	237.2	AF		dipeptidyl peptidase IV inhibitor; ACE inhibitor	17.0	4.3	33.7	14.6	2.9	4.4	0.9	2.8
14.83	231.2	L (I) V	v/21k	glucose uptake stimulating peptide (IV, LV); dipeptidyl peptidase IV inhibitor (LV)	8.1	12.0	17.7	11.8	1.9	8.7	0.7	7.4
14.9	634.3	VSTDVN	21k		0.3	0.3	11.8	5.1	0.3	0.9	0.0	0.5
14.95	229	PI (L)		dipeptidyl peptidase IV inhibitor (PI, PL); ACE inhibitor (PL)	11.8	7.4	23.4	18.9	3.5	8.2	1.3	8.8
15.1	431	unk			7.5	9.2	52.5	28.4	2.5	2.7	0.7	1.2
15.24	487.3	ANSPV	21k		2.6	2.7	27.5	25.9	1.5	4.9	0.4	2.9
15.32	295	EF		CaMPDE inhibitor; Renin inhibitor (HYPOTENSIVE)	6.3	4.4	23.1	11.9	1.3	2.8	0.3	2.7
15.44	838.4	DEEGNFK	v		0.1	0.0	2.4	0.0	0.1	0.0	0.0	0.0
15.77	231.2	VI (L)	v/21	dipeptidyl peptidase IV inhibitor (VI, VL), glucose uptake stimulating peptide (VL)	44.6	12.5	17.7	11.8	6.7	11.6	2.3	6.8
15.8	488.3	GAGGGGL	v		4.8	3.6	28.4	12.5	0.6	0.8	0.1	0.5
16.06	296.2	YN		dipeptidyl peptidase IV inhibitor, ACE inhibitor	6.9	7.3	14.8	13.1	1.7	3.7	0.7	1.8
16.25	263.3	FP		dipeptidyl peptidase IV inhibitor; ACE inhibitor	3.8	5.3	10.7	7.5	0.8	3.1	0.3	3.5
16.37	379.9 (757.4)	ASKDQPL	v		1.3	0.2	4.4	0.7	1.4	0.6	0.3	0.3
16.8	265.3	FV	v/21k		7.6	6.6	30.2	18.7	2.3	4.7	1.0	3.0
17.16	245.2	II IL LL LI		ACE inhibitor (IL), glucose uptake stimulating peptide; dipeptidyl peptidase IV inhibitor	5.3	7.1	9.1	6.8	0.8	7.2	0.2	7.8
17.3	276.1	AW	21k	ACE inhibitor; antioxidant; dipeptidyl peptidase IV inhibitor	8.6	5.7	29.1	13.9	1.1	1.9	0.1	0.8
17.86	360.3	VLE	V		0.1	4.4	0.6	0.0	0.5	0.8	0.1	0.7
18.1	265.2	VF	V	ACE inhibitor; dipeptidyl peptidase IV inhibitor	19.6	10.7	56.6	27.8	3.8	6.8	1.2	4.6
18.19	393.3	FLN/SSIS	V/21k		2.3	0.6	17.6	8.1	1.2	1.4	0.3	0.5
18.19	245.2	II IL LL LI		ACE inhibitor (IL), glucose uptake stimulating peptide; dipeptidyl peptidase IV inhibitor	4.1	19.0	0.0	10.8	0.8	7.5	0.3	7.5
18.22	710.4	DEEGNF	V		1.1	0.1	20.8	0.6	0.8	0.2	0.1	0.3
18.57	243.2	Pyroglu-LEU			9.3	13.9	29.8	35.2	2.7	6.8	1.1	5.6
18.69	245	II IL LL LI		ACE inhibitor (IL), glucose uptake stimulating peptide; dipeptidyl peptidase IV inhibitor	19.6	15.8	0.0	9.9	4.1	11.8	1.3	8.5
18.93	690.3	NGKGTIT	V		0.2	0.1	5.5	2.0	0.1	0.1	0.0	0.1
19.05	328	VPI			35.7	25.5	133.3	72.4	1.9	7.7	0.6	5.6
19.53	243.2	Pyroglu-ILE			6.5	7.9	24.2	17.2	2.5	4.4	1.0	3.9
19.7	245.2	II IL LL LI		ACE inhibitor (IL), glucose uptake stimulating peptide; dipeptidyl peptidase IV inhibitor	7.2	9.3	20.2	12.8	1.8	9.8	0.5	8.8
19.85	534.3	PGDVF	V		0.0	0.0	17.5	0.0	0.6	0.1	0.2	0.0
19.89	933.6	DSKDDVVR	21k		0.1	0.0	2.8	0.0	0.1	0.1	0.0	0.1
20.16	564.3	RRSF	V		0.6	0.0	5.5	1.1	0.2	0.1	0.1	0.2
20.22	279.3	L (I) F		ACE inhibitor	7.1	7.7	27.3	13.3	1.6	4.3	0.8	3.2
20.28	563.3	DEEGN	V		3.9	0.0	21.8	0.0	0.8	0.3	0.1	0.3
20.32	360.3	EVL	V		2.6	0.0	4.4	3.4	0.3	0.3	0.1	0.2
20.58	862.5	SSISGAGGGGL	21K		0.3	0.1	12.5	3.8	0.3	0.7	0.1	0.4
21	437.3	GDVF	V		0.6	0.2	5.5	0.0	0.6	0.1	0.2	0.2
21.16	279.3	L (I) F		ACE inhibitor	24.5	18.3	74.7	60.0	4.9	19.1	1.8	11.8
21.19	600.4	KDQPL	V		0.8	0.0	29.4	0.0	0.9	0.0	0.2	0.2
21.31	277	Pyroglu-PHE			8.8	9.0	24.1	22.0	3.2	4.2	1.3	2.2
21.4	380.2	DVF	V		8.0	0.1	25.0	1.9	1.6	0.3	0.6	0.2
22.09	279.3	FL (I)		dipeptidyl peptidase IV inhibitor (FL)	3.6	3.3	30.2	0.0	2.3	4.2	0.7	3.2
22.41	279.3	FL (I)	v		9.4	2.2	30.2	0.0	2.3	3.2	0.7	1.9
23	621.5	SPGDVF	V		0.4	0.0	37.7	0.5	1.6	0.1	0.5	0.0
23.96	408.3	IEF	21K		1.9	0.0	17.9	0.4	1.8	0.1	0.5	0.0
25.69	603.3 (1204)	SNADSKDDVVR	21k		0.0	2.3	0.3	0.0	0.0	2.2	0.0	4.1
26.31	789.6	TVWRLD	21K		0.0	0.6	0.2	0.1	0.0	0.0	0.0	0.3
27.59	601.3	NNKPE			0.0	3.6	0.7	0.0	0.0	1.3	0.0	1.7
27.92	747.5	NGTPVIF	21K		0.6	0.0	12.7	0.4	0.1	0.0	0.0	0.1
28.13	533.1 (1063.5)	DEEGNFKIL	V		0.0	0.0	2.3	0.4	0.2	0.1	0.0	0.0
28.54	902.6	APLSPGDVF	V		0.0	0.0	1.2	0.0	0.3	0.0	0.1	0.0
29.76	820.5	DNEWAW	21K		0.1	0.0	2.2	0.0	0.0	0.1	0.0	0.2
				**Total peptides**	**427.9**	**313.2**	**1348.7**	**655.0**	**100.0**	**242.3**	**33.1**	**203.2**

**Table 2:** ACE inhibitory activity of cocoa samples digestates, reported as percentage of inhibition respect to ACE maximum activity (relative to digestion blank).

Solution	Concentration (mg/mL)	Esteemed Peptide Concentration (μg/mL)	I%
Theobromine	0.3	0	19 ± 5
Epicatechin	0.2	0	20 ± 5
Amino acid mixture	5	0	5 ± 4
Cocoa bean digestate	50	33	95 ± 4
Cocoa paste digestate	50	12	63 ± 10
Chocolate digestate	50	10	75 ± 8

**Table 3:** Computational in silico results obtained for the analyzed peptides sequences, presented as HINT (Hydrophatic INTeraction) scores and expected activity of peptides under analysis.

	Cdominio (cutoff 260)		Ndominio (cutoff 540)		TOT
**Compound**	**HS**	**Interaction**	**HS**	**Interaction**	
FE	2995	Positive	2443	positive	Active
FV	2494	Positive	2776	Positive	Active
II	2042	Positive	2380	Positive	Active
IL	1935	Positive	2262	Positive	Active
LI	2053	Positive	2045	Positive	Active
LL	1792	Positive	2062	Positive	Active
LY	3110	Positive	2709	Positive	Active
PI	1811	Positive	1717	positive	Active
PL	1992	Positive	1550	Positive	Active
QE	2091	Positive	1466	Positive	Active
SPV	1171	Positive	2075	Positive	Active
VEL	757	Positive	2160	Positive	Active
VF	2406	Positive	2366	Positive	Active
VI	1475	positive	2364	positive	Active
VL	1584	positive	2212	positive	Active
VPI	1258	positive	1707	positive	Active
VPL	1200	positive	1203	positive	Active
LLDR	−913	negative	1224	positive	Active
QLGN	799	positive	413	negative	Active
